# Convolutional neural networks for accurate real-time diagnosis of oral epithelial dysplasia and oral squamous cell carcinoma using high-resolution in vivo confocal microscopy

**DOI:** 10.1038/s41598-025-86400-5

**Published:** 2025-01-20

**Authors:** Rishi S. Ramani, Ivy Tan, Lindsay Bussau, Lorraine A. O’Reilly, John Silke, Christopher Angel, Antonio Celentano, Lachlan Whitehead, Michael McCullough, Tami Yap

**Affiliations:** 1 Melbourne Dental School, University of Melbourne, Level 5, 720 Swanston Street, Carlton, Melbourne, VIC 3053 Australia; 2 Optiscan Imaging Ltd, Mulgrave, VIC Australia; 3 Walter and Eliza Hall Institute, Melbourne, VIC Australia; 4 Peter MacCallum Cancer Centre, Melbourne, VIC Australia; 5 Department of Medical Biology, University of Melbourne, Melbourne, VIC Australia

**Keywords:** Oral cancer, Early diagnosis, Digital microscopy, Deep learning, Computer science, Cancer imaging, Cancer screening, Oral cancer

## Abstract

Oral cancer detection is based on biopsy histopathology, however with digital microscopy imaging technology there is real potential for rapid multi-site imaging and simultaneous diagnostic analysis. Fifty-nine patients with oral mucosal abnormalities were imaged in vivo with a confocal laser endomicroscope using the contrast agents acriflavine and fluorescein for the detection of oral epithelial dysplasia and oral cancer. To analyse the 9168 images frames obtained, three tandem applied pre-trained Inception-V3 convolutional neural network (CNN) models were developed using transfer learning in the PyTorch framework. The first CNN was used to filter for image quality, followed by image specific diagnostic triage models for fluorescein and acriflavine, respectively. Images were categorised based on a histopathological diagnosis into 4 categories: no dysplasia, lichenoid lesions, low-grade dysplasia and high-grade dysplasia/oral squamous cell carcinoma (OSCC). The quality filtering model had an accuracy of 89.5%. The acriflavine diagnostic model performed well for identifying lichenoid (AUC = 0.94) and low-grade dysplasia (AUC = 0.91) but poorly for identifying no dysplasia (AUC = 0.44) or high-grade dysplasia/OSCC (AUC = 0.28). In contrast, the fluorescein diagnostic model had high classification performance for all diagnostic classes (AUC range = 0.90–0.96). These models had a rapid classification speed of less than 1/10th of a second per image. Our study suggests that tandem CNNs can provide highly accurate and rapid real-time diagnostic triage for in vivo assessment of high-risk oral mucosal disease.

**Supplementary Information:**

The online version contains supplementary material available at 10.1038/s41598-025-86400-5.

## Introduction

Oral cancer is a deadly disease and detection at an early stage is the most effective means to improve survival, morbidity, duration of treatment, psychological outcomes and quality of life^1^. Currently, two-thirds of patients with oral cancer are diagnosed at an advanced stage of the disease with a 5-year survival rate of only 50%^2,3^. A short time interval from first symptoms to diagnosis and subsequent referral to a specialist significantly improves patient survival^4^.

An important early indicator of oral cancer may involve the identification and detection of oral potentially malignant disorders (OPMD). OPMDs are a group of mucosal disorders that may precede oral squamous cell carcinoma (OSCC). Specifically, individuals with diagnosed OPMDS have an increased susceptibility of developing oral cancer^5^. Currently, histological examination of biopsy tissue has been the primary means to identify pathological changes in the oral epithelium indicative of an increased risk of malignant transformation, termed as oral epithelial dysplasia (OED). This approach can be invasive, painful, susceptible to specimen handling errors and subjective to human observation and interpretation^6^. An alternative method for early detection of OED and OSCC is confocal microscopy, an optical imaging method that achieves sub-cellular resolution for in vivo imaging at an advanced frame rate^7^.

One of the challenges in the field of digital bio-imaging is that modern microscopes, whilst having the ability to produce a large volumes of image data, still require manual curation by clinicians to ascertain meaningful data. Such manual image quality sorting could represent a future bottleneck in clinical applications of image analyses pipelines emerging from this technology. In addition, real-time in vivo microscopic imaging introduces elements such as mobile intraoral tissues, saliva, air or debris that may lead to images of poor diagnostic quality. Artificial intelligence (AI) methods are being developed to address these concerns in medical imaging^8^.

Deep learning is a subfield of machine learning and AI that involves complex algorithmic models to learn insights from data to make predictions^9^. Convolutional neural networks (CNN) are the best performing deep learning models for visual problems. The Inception_v3 CNN architecture is one such model that performed exceptionally well at the ImageNet Large Scale Visual Recognition Challenge in 2015, having a top-5 error rate of 3.58%^10^. Model architectures such as Inception_v3 can be optimized for image classification tasks other than what they were created for by using transfer learning^11^. CNN models implement hyperparameters while training that are selected by the practitioner, unlike other parameters and coefficients that are learned from actual data^12^. Some hyperparameters, such as number of epochs (number of times the model processes the entire training data) and learning rate (variable controlling the rate of adjustment of model parameters to reduce error) can be selected by a practitioner using heuristics or optimised using algorithms^13^.

The aim of the present research was to develop, train, and test convolutional neural networks trained on captured human oral in vivo confocal micrographs for quality filtering and real-time detection of histopathological classification including OSCC. This combination of technologies has the potential to positively impact patient outcomes and quality of life by reducing the need for invasive biopsies. Furthermore, the remote application capabilities and the potential for this expertise to extend beyond just dental practitioners could enhance accessibility and expand the reach of this transformative diagnostic tool.

## Methods

This study demonstrates a protocol for CNN development, training, and testing while incorporating hyperparameter optimization, and k-fold cross validation for improved prediction performance. The study design aligns with the STARD checklist for reporting diagnostic accuracy and the WHO-ITU checklist for artificial intelligence research in dentistry^14,15^.

### Data acquisition and processing

This study was approved by the Human Research Ethics Committee within the Medicine, Dentistry and Health Sciences Faculty at the University of Melbourne (Ethics ID: 1955205) and Dental Health Services Victoria (RRG: 341) and conducted in accordance with the Declaration of Helsinki. Written informed consent was obtained from all patients.

#### Participants and image acquisition

**Fig. 1:**
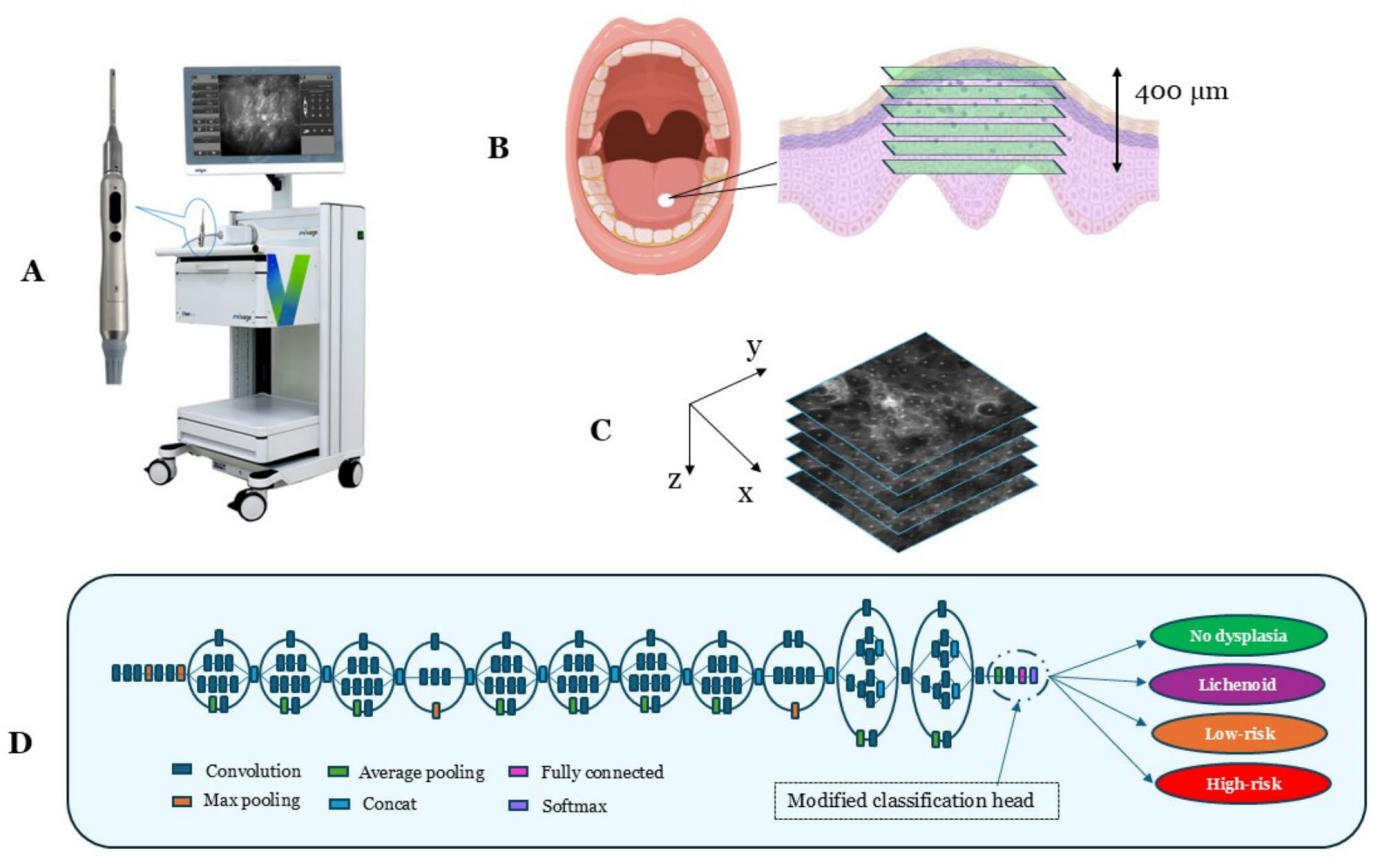
In vivo confocal microscopy image capture and CNN architecture. (**A**) InVivage confocal endomicroscope (Optiscan Imaging Ltd, Australia) with handheld probe (inset) (**B**) Captured image stacks in an en face orientation extended up to 400 μm depth into the oral epithelium, (**C**) Schematic of a captured of image stack along the z axis, (**D**) The modified Inception_v3 CNN architecture for the PMAC diagnostic models.

Fifty-nine patients attending the Oral Medicine Department of the Royal Dental Hospital of Melbourne (Melbourne, Australia) for assessment of oral mucosal abnormalities were imaged between 08/12/2020 to 13/03/2022 using the InVivage in vivo confocal laser endomicroscope (Optiscan Imaging Ltd, Australia) (Fig. 1A). Imaging was conducted with topically applied imaging agents, 0.1% fluorescein and 0.1% acriflavine^16^. A total of 9168 images were obtained from this cohort across oral sites of the tongue, buccal mucosa, gingiva & vestibule, soft palate, hard palate, and floor of the mouth (IT, TY, & MMc). Scalpel biopsy followed by histopathological diagnosis were completed following assessment by the treating oral medicine specialist (TY, MMc). The site location of the biopsy was selected by the treating specialist before confocal microscope imaging.

#### Imaging and processing hardware

The InVivage confocal microscope has a lateral and axial resolution of 0.55 μm and 5.1 μm respectively with a field-of-view of 475 × 475 μm and a z-axis depth focus range of 400 μm. The imaging plane orientation was parallel to the surface of the tissue with the z-axis oriented along the tissue depth (Fig. 1A, B and C)^7^. The training and testing of CNNs with image analysis was carried out using an NVIDIA RTX 3050 graphics processing card with an AMD Ryzen 5000 series processor with 16 GB random access memory. The clinical imaging protocol has been described previously by Yap et al., 2023^16^. All references to ‘images’ in this study allude to image frames/slices captured by the in vivo confocal microscope along the z-axis when recording as an image stack (Fig. 1B and C).

#### Diagnostic categories

**Fig. 2:**
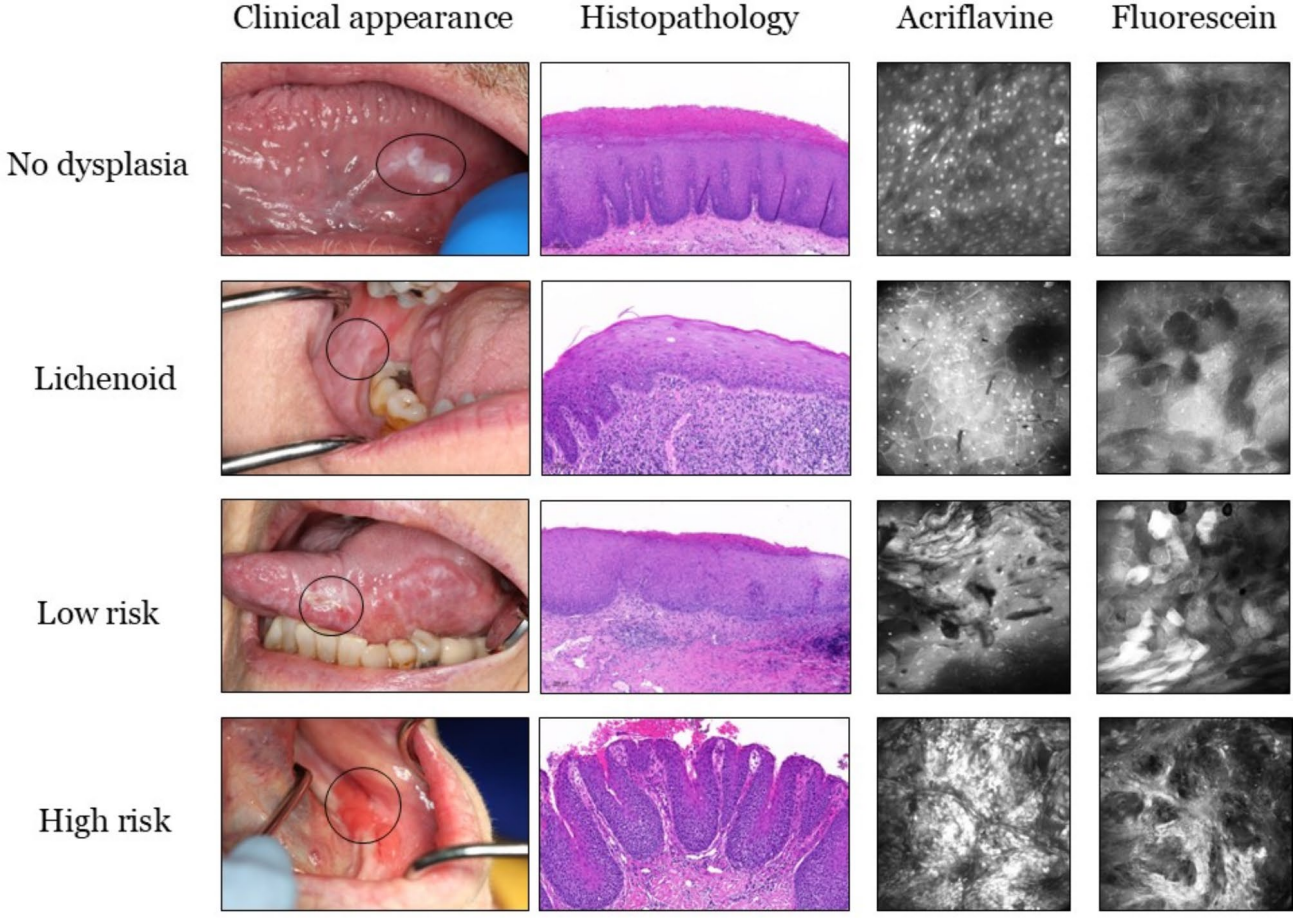
Panel of image examples from all 4 diagnostic classes. Examples of clinical photographs, histopathology slides, acriflavine and fluorescein confocal micrographs from one participant each belonging to ‘no dysplasia’, ‘lichenoid’, ‘low-risk’, and ‘high-risk’. The lesions identified in the clinical images are marked using a black circle. ‘no dysplasia’ example depicts a case of hyperkeratosis and hyperplasia (no dysplasia) on the lateral tongue margin. ‘lichenoid’ example depicts a case of lichenoid inflammation on the buccal mucosa. ‘low-risk’ example depicts a case of mild dysplasia lateral tongue margin. ‘high-risk’ example depicts a case of focal moderate dysplasia on the buccal gingiva and vestibule.

The ‘No dysplasia’ category included lesions that histopathologically showed no signs of OED. Due to the indistinguishable separation of oral lichen planus (OLP) and oral lichenoid lesions (OLL) by histopathological features, such lesions were considered as a separate ‘Lichenoid’ category in this study^17–19^, as clearly distinct from OED. The lesions displaying minor atypia (focal epithelial changes), verrucous hyperplasia or low grade dysplasia were categorised as ‘Low-risk’ lesions due to their potential for malignant transformation. High grade dysplasia and OSCC lesions were categorised as ‘High-risk’. The binary dysplasia grading for OPMDs as proposed by Kujan et al. (2006) and adopted by the WHO was used in this study^20^. The binary system categorises lesions based on the overall number of observed cytological and architectural features (WHO 2005 grading system^20^) (Fig. 2).

### Convolutional neural network (CNN) development

#### Development framework

Neural networks described in this study were developed within the PyTorch (ver. 2.1.0) framework using Python 3 programming language (ver. 3.11.5)^21,22^. Python libraries of Numpy (ver. 1.24.3) and Pandas (ver. 2.1.1.) were used for performing numerical data analysis and the Sci-kit learn library (ver. 1.3.0.) was used for machine learning applications^23–25^. The CNN architecture in this study was a modified Inception_v3 architecture where the final classification layer was replaced by a new fully connected layer for the classes required in this study using transfer learning (Fig. 1D). Before the images were loaded into a PyTorch environment they underwent pre-processing steps which included converting file type from DICOM to TIFF and resizing images from 1024 × 1024 to 299 × 299 pixels, based on Inception-V3 input requirements using the open-source image analysis software Fiji (Image J)^26^.

#### Hyperparameter optimization

An optimization process was conducted to identify the best adjustable parameters for the deep learning models applied to our dataset. A grid search approach was employed for hyperparameter optimization across a search space of values for number of epochs and learning rate ranging from ‘5’, ‘10’, ‘15’, ’20’, ‘25’, and ‘30’ epochs and learning rates of ‘0.001’, ‘0.01’, ‘0.1’, respectively^27^. The training dataset was split into training and validation sets using k-fold cross validation where k = 5. This resulted in each cross-validation fold having 80% of the data constituting the training set and 20% the validation set, with the data being shuffled across all the folds. One models were trained for each combination of epoch and learning rate for each cross-validation fold.

Three unique types of neural network models were trained to perform two separate tasks of quality filtering and diagnostic triage for images from each of the contrast agents, acriflavine and fluorescein. The quality filter model, named the Quality Micrograph Refiner (QMR), was applied to images obtained using both imaging agents and preceded an imaging agent specific diagnostic triage model. The diagnostic triage models were named the Fluorescein Pathologic Micrograph Allocation CNN (FPMAC) and Acriflavine Pathologic Micrograph Allocation CNN (APMAC), respectively. A total of 270 CNN models across all combinations of epochs, learning rates and cross validation folds for both contrast agents were developed, trained, and tested in PyTorch.

#### Performance assessment

The performance metrics used to assess all CNN models in this study were accuracy (%), sensitivity, specificity, precision, and F1 score. The accuracy results across all folds of the 5-fold cross-validation were averaged to estimate the most optimum hyperparameter combination. All trained models were ranked based on an aggregation of ranks for the 5 metric scores calculated. The overall rank was calculated by ranking the aggregate rank scores for each hyperparameter combination.

The performance results of all trained models were categorized separately for each class. Among models from individual folds of cross validation overall aggregate ranks across all 5 metrics and for all classes (one vs. all) were calculated where the rank #1 model represents the best performing model for predicting all classes. Performance metrics for each diagnostic category was calculated individually using a one vs. all approach. Additionally, receiver operator characteristic (ROC) curves were plotted and the area under the curve (AUC) calculated for each diagnostic class vs. all for both contrast agent diagnostic CNNs. All statistical analyses were performed using the Python sci-kit learn library^24^.

### Quality micrograph refiner (QMR) model creation

The QMR CNN was designed to discard confocal micrograph images captured using either contrast agent that were not of diagnostic quality. The criteria used for including images of sufficient quality necessitated the presence of visible oral epithelial cell borders or oral epithelial cell nuclei in focus of the microscope lens. Based on the criteria, the images containing major artifacts, imaging errors, or featureless zones that covered equal to or more than 75% of the field of view of the confocal micrograph were not considered to be of diagnostic quality.

For training, a dataset of 800 images representing all sites and contrast agents taken from 30 study participants were manually annotated for diagnostic quality by blind screening, followed by a consensus decision between three investigators (IT, RR, TY and MMc). To test the QMR a test dataset was constructed with 400 previously unseen images from the same 30 participants. These test images were also manually evaluated and annotated by blind screening and again followed by a consensus decision between three investigators (RR, TY, and MMc).

### Division of dataset into diagnostic categories for PMAC

**Fig. 3:**
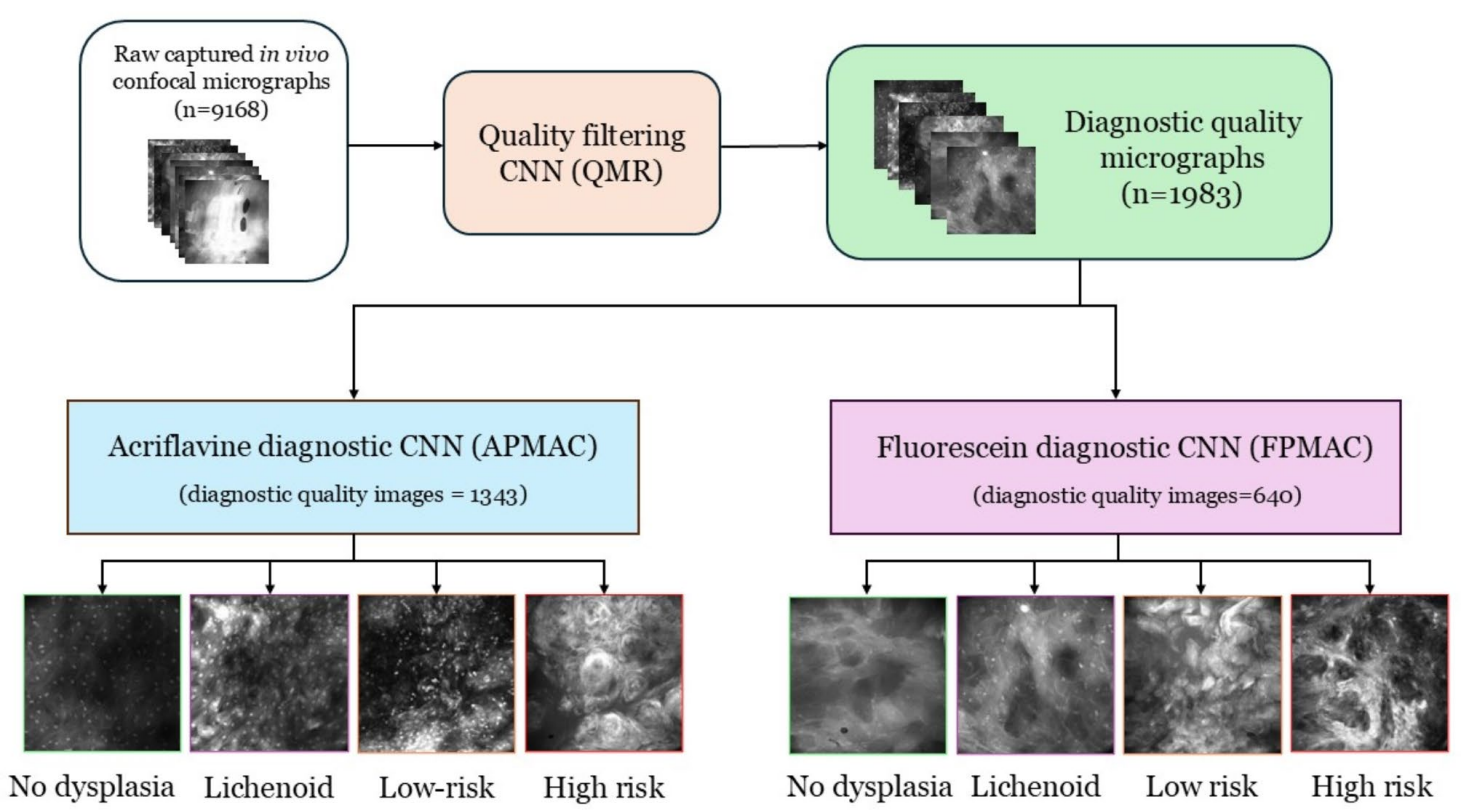
Flow diagram for workflow of CNN quality and diagnostic analysis of in vivo captured micrographs. This flow diagram shows the processing workflow of raw in vivo images captured by the InVivage confocal endomicroscope. The raw images were first used to develop the quality filtering CNN (QMR). Following this the QMR was used to filter the entire dataset (*n* = 9168). The diagnostic quality images after filtering (*n* = 1983) were divided based on contrast agent used and assigned to the acriflavine diagnostic CNN (APMAC) (*n* = 1343) and fluorescein diagnostic CNN (FMPAC) (*n* = 640). These diagnostic CNNs were developed using these images to classify them into ‘no dysplasia’, ‘lichenoid’, ‘low-risk’, and ‘high-risk’.

The trained QMR was applied to all 9168 available images, resulting in 1983 diagnostic quality images usable for analysis, which were subsequently used for training and testing the PMAC models (Supplementary Table 1). This diagnostic quality dataset of 1343 Acriflavine and 640 Fluorescein images were divided into 4 categories of ‘No dysplasia’, ‘Lichenoid’, ‘Low-risk’ and ‘High-risk’ (Fig. 3) across 80% training and 20% test images (Table 1, Supplementary Table 2). These images were annotated by labelling each confocal microscopy image using the diagnostic categories based on the histopathology standard reference.

**Table 1:** Image distribution for Acriflavine and Fluorescein training and test image sets.

Diagnosis categories	Acriflavine training (80%)	Acriflavine test (20%)	Fluorescein training (80%)	Fluorescein test (20%)
No dysplasia	449	108	151	35
Amalgam tattoo	25	6	0	0
Chronic inflammation	16	3	8	2
Denture associated hyperplasia	24	5	4	1
Fibroepithelial polyp	5	1	8	1
Focal papillomatosis	38	9	7	1
Hyperplasia & hyperkeratosis	325	82	119	29
Squamous papilloma	5	1	5	1
Verruciform xanthoma	10	2	0	0
Lichenoid	**275**	**68**	**151**	**37**
Oral lichenoid lesion	175	43	103	25
Oral lichen planus	100	25	48	12
Low-risk	**286**	**69**	**127**	**31**
Atypia	42	10	0	0
Low grade dysplasia	132	32	91	22
Verrucous hyperplasia	112	27	36	9
High-risk	**71**	**17**	**86**	**22**
High grade dysplasia	60	15	84	21
OSCC	11	2	2	1
Grand total	1081	262	515	125

From the images of diagnostic quality, 80% were randomly categorised as training images, with the remaining 20%, were utilised for testing of both contrast agents. Within the training set, 5-fold cross validation was carried out to assess 5 different combinations of learning-validation sets, which were also divided by a ratio of 80:20.

## Results

A total of 270 CNN variants were developed, trained, and tested in this study across ~ 30 h of training and testing. This included 90 models for each of the 3 CNN tasks: quality micrograph refiner (QMR), Acriflavine diagnostic CNN (APMAC) and Fluorescein diagnostic CNN (FPMAC) across all hyperparameter combinations with cross validation folds.

### Performance results of QMR CNN

The best hyperparameter combination for CNN performance on the test dataset was 15 epochs with a learning rate of 0.01 (Table 2). The averaged performance metrics of this parameter combination across all 5 cross validation folds had an accuracy of 88.1% with a sensitivity of 0.78, specificity of 0.94, precision of 0.90, and a F1 score of 0.83.

**Table 2:** Averaged test classification performance of the QMR model across all cross-validation folds for all hyperparameter combinations.

Epochs	LR		Accuracy	Sens.		Spec.		Precision		F1 score		Overall rank	
5	0.001		64.0%	0.07		1.00		0.95		0.13		12	
5	0.01		86.0%	0.75		0.93		0.87		0.80		5	
5	0.1		82.3%	0.75		0.87		0.79		0.76		18	
10	0.001		73.1%	0.31		0.99		0.97		0.47		11	
10	0.01		85.5%	0.69		0.96		0.91		0.78		2	
10	0.1		83.1%	0.74		0.89		0.81		0.77		14	
15	0.001		78.5%	0.51		0.96		0.88		0.64		17	
15	**0.01**		**88.1%**	**0.78**		**0.94**		**0.90**		**0.83**		**1**	
15	0.1		83.7%	0.72		0.91		0.84		0.77		12	
20	0.001		83.0%	0.62		0.96		0.91		0.74		9	
20	0.01		85.1%	0.70		0.95		0.89		0.78		7	
20	0.1		83.5%	0.66		0.94		0.88		0.75		15	
25	0.001		84.4%	0.68		0.95		0.89		0.77		8	
25	0.01		85.2%	0.70		0.94		0.89		0.79		6	
25	0.1		83.7%	0.68		0.93		0.87		0.76		16	
30	0.001		85.6%	0.72		0.94		0.89		0.79		3	
30	0.01		85.3%	0.71		0.95		0.89		0.79		3	
30	0.1		83.8%	0.76		0.88		0.81		0.78		9	

LR = Learning rate, Sens. = Sensitivity, Spec. = Specificity

The best ranked individual model with this hyperparameter combination based on ranking performance metrics was chosen as the QMR model. This model had an accuracy of 89.5% with a sensitivity of 0.81, specificity of 0.95, precision of 0.91, and a F1 score of 0.86, while taking 0.03 seconds to analyse each image.

The results of testing the best QMR model were extracted for each intraoral site imaged. Imaging quality varied according to intraoral site, with the highest F1 scores from the gingiva and vestibule (0.90) and floor of the mouth (0.91) sites. Micrographs with the poorest identification F1 score were from images of the hard palate (0.71) (Table 3).

The QMR model applied to the entire dataset of 5359 acriflavine images and 3809 fluorescein images retained 1343 (25.06%) and 640 (16.80%) diagnostic quality images respectively. The highest number of acriflavine images were retained for the tongue (*n* = 554, 25.61%) and the lowest for hard palate (*n* = 21, 14.58%). The highest number of fluorescein images were also retained for the tongue (*n* = 230, 15.59%) and the lowest for hard palate (*n* = 2, 2.33%) (Supplementary Table 1).

### Training and testing Acriflavine PMAC (APMAC)

**Fig. 4:**
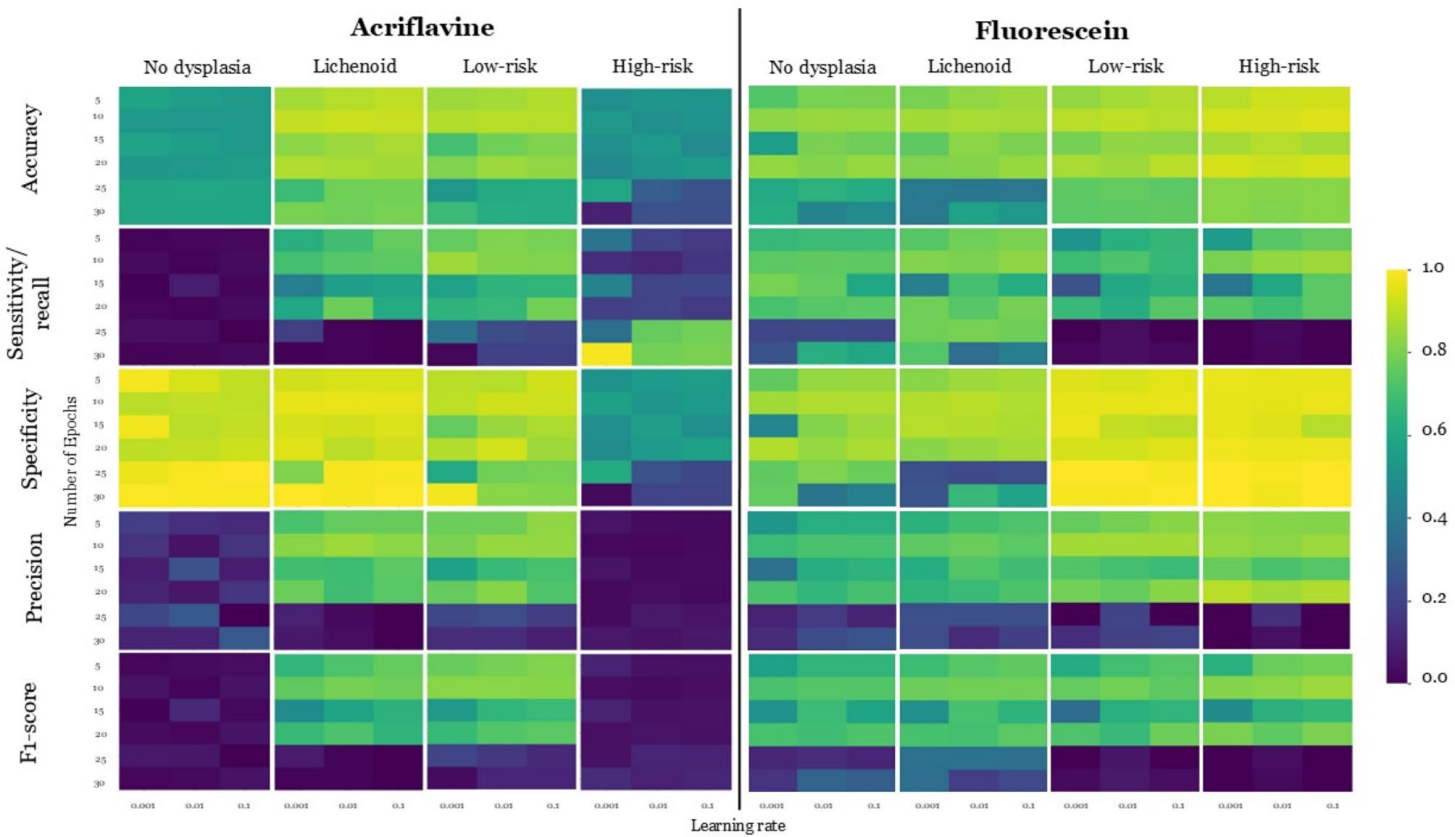
Acriflavine (APMAC) and fluorescein diagnostic model (FPMAC) hyperparameter optimisation results. Heat maps for with colour coding from blue (0) to yellow (1.0) for validation metrics of accuracy, sensitivity, specificity, precision and F1 score, indicate the performance of different hyperparameter combinations across number of epochs and learning rate during APMAC and FPMAC training to ascertain the optimal hyperparameter combination for each model to maximise performance.

**Fig. 5:**
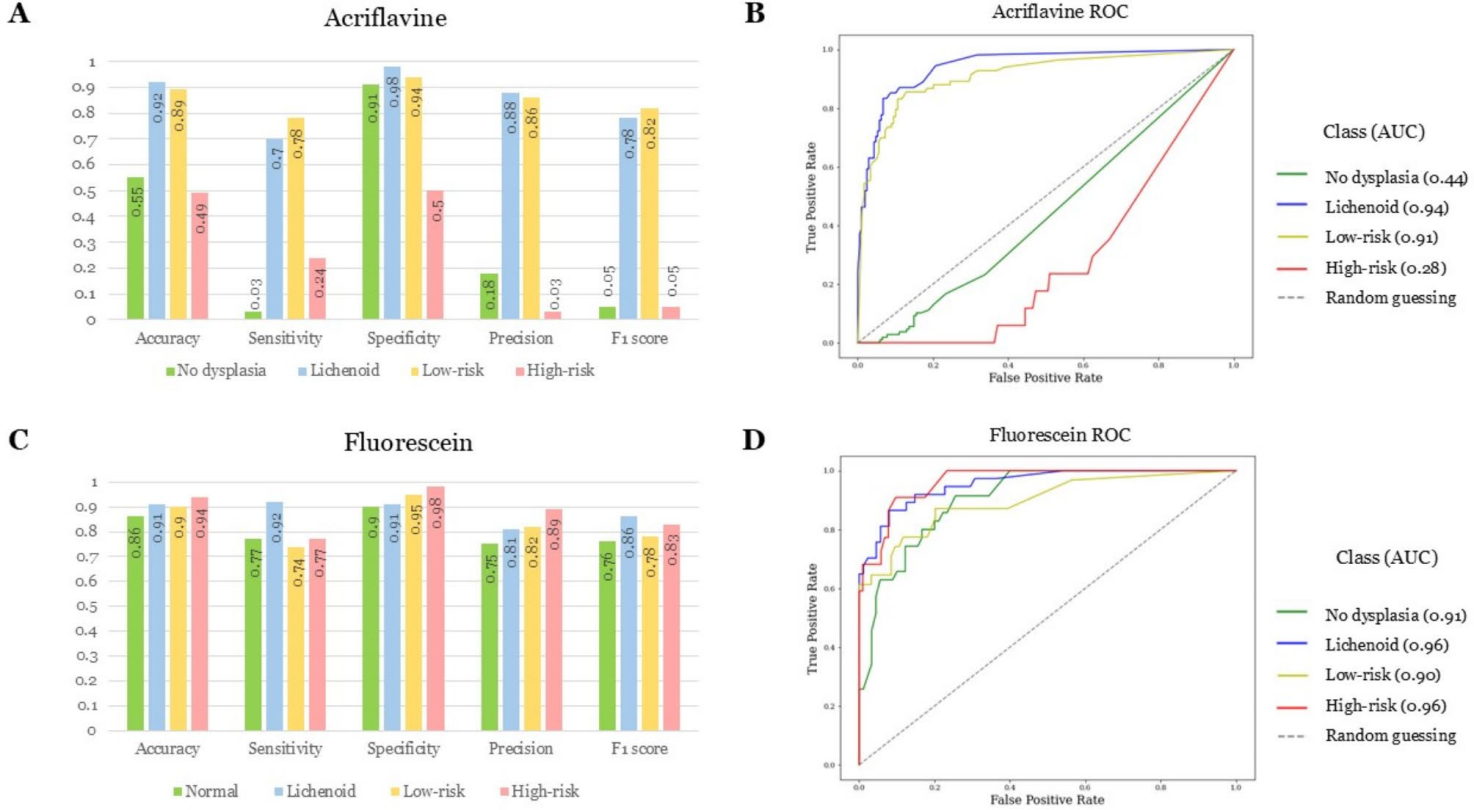
Classification performance test results for trained acriflavine and fluorescein diagnostic models (APMAC & FPMAC). (**A**) Graphical representation of test results for the APMAC across validation metrics: accuracy, sensitivity, specificity, precision and F1 score for detecting no dysplasia (green), lichenoid (blue), low-risk (amber) and high-risk (red); (**B**) Receiver operator curves (ROC) curves for the trained APMAC model across all four diagnostic classes with their area under the curve (AUC); (**C**) Graphical representation of test results for the FPMAC across validation metrics: accuracy, sensitivity, specificity, precision and F1 score for detecting no dysplasia (green), lichenoid (blue), low-risk (amber) and high-risk (red); (**D**) Receiver operator curves (ROC) curves for the trained FPMAC model across all four diagnostic classes with their area under the curve (AUC).

The best hyperparameter combination across all 90 acriflavine models trained and tested across all diagnostic categories (no dysplasia, lichenoid, low-risk, high-risk) that had the best overall sensitivity, specificity, precision, and F1 score was 30 epochs, with a learning rate of 0.001 (Fig. 4). This model was therefore selected to be the APMAC model. Utilising this model the F1 scores for lichenoid and low-risk lesions was high at 0.78 and 0.82, respectively. Whereas the no dysplasia and high-risk classification F1 scores were both 0.05 (Fig. 5A). The APMAC model F1 scores were highest for floor of the mouth and buccal mucosa at 0.83 and 0.77 respectively. While the lowest F1 scores were for hard palate and gingiva & vestibule at 0 and 0.17 respectively (Table 3).

**Table 3:** Performance of all CNN models across different intra-oral imaging sites.

CNN Model		Locations			Accuracy (%)		Sensitivity		Specificity		Precision		F1 score	
QMR		Buccal mucosa			86		0.78		0.94		0.93		0.85	
		Floor of mouth			90.48		0.86		0.96		0.97		0.91	
		Gingiva & vestibule			93.48		0.93		0.94		0.87		0.9	
		Hard palate			87.65		0.63		0.95		0.8		0.71	
		Soft palate			90		0.8		1		1		0.89	
		Tongue			92		0.84		0.96		0.9		0.87	
Acriflavine PMAC		Buccal mucosa			75.44		0.75		0.90		0.78		0.77	
		Floor of Mouth			80		0.80		0.95		0.88		0.83	
		Gingiva & Vestibule			18.06		0.18		0.75		0.32		0.17	
		Hard palate			0		0.00		0.00		0.00		0.00	
		Soft palate			50		0.50		0.50		0.50		0.50	
		Tongue			46.67		0.47		0.84		0.49		0.44	
Fluorescein PMAC		Buccal mucosa			78.57		0.79		0.89		0.85		0.81	
		Floor of Mouth			87.50		0.88		1.00		1.00		0.93	
		Gingiva & Vestibule			72.73		0.73		0.92		0.87		0.76	
		Soft palate			100		1.00		1.00		1.00		1.00	
		Tongue			61.70		0.62		0.87		0.65		0.62	

The ROC curves for the APMAC model (Fig. 5B) further depict the disparity in the model’s ability to detect lichenoid (AUC = 0.94) and low-risk lesions (AUC = 0.91) compared to no dysplasia (AUC = 0.44) and high-risk (AUC = 0.28) lesions. The best ranked APMAC model took 16.60 seconds to classify all 262 test images at the rate of 0.06 seconds per image.

### Training and testing Fluorescein PMAC (FPMAC)

The best hyperparameter combination across all 90 fluorescein models trained and tested in all diagnostic categories (no dysplasia, lichenoid, low-risk, high-risk) that had the best overall sensitivity, specificity, precision, and F1 score was 25 epochs, with a learning rate of 0.001 (Fig. 4). This model was chosen to be the FPMAC. This model had highest F1 scores for lichenoid (0.86) and low-risk (0.83) classes with scores of 0.76 and 0.78 for no dysplasia and high-risk, respectively (Fig. 5C). The FPMAC model F1 scores were highest for soft palate and floor of the mouth at 1 and 0.93 respectively. While the lowest F1 scores were for tongue and gingiva & vestibule at 0.62 and 0.76 respectively (Table 3).

The AUC for the ROC curves for no dysplasia (AUC = 0.91), lichenoid (AUC = 0.96), low-risk (AUC = 0.90), and high-risk (AUC = 0.96) demonstrate effectiveness of the model at identifying all the classes (Fig. 5D). This FPMAC model took 5.59 seconds to classify all 125 test images at the rate of 0.04 seconds per image.

## Discussion

The present study uniquely represents the use of hyperparameter optimisation and cross validation to develop highly accurate and rapid deep learning classification models for the real time prediction of oral mucosal lesions based on fluorescence in vivo confocal microscopy images.

While biopsy histopathology represents the standard of care for definitive diagnosis of oral mucosal conditions, the acquisition of these biopsies under local anaesthesia often leads to patient anxiety and post-procedural oral discomfort. Assumptions made in histopathology based on excised tissue extend to attributing the observations in the sample to the entire abnormal mucosal area. To address these limitations, non-invasive technologies are being advanced to offer high-resolution, rapid, and multi-site assessments of the oral mucosa, surpassing the diagnostic capabilities of standard white-light examination. Folmsbee et al. (2018) used a CNN called AlexNet to identify histopathology oral cancer images with an accuracy of 96.44%^28^. Another recent study applied CNNs to histopathology slide images for oral cancer detection showed an accuracy of 92.15%^29^. While displaying promising results, this approach does not aid in limiting the use of histopathology in the diagnostic process. CNNs have also been used to identify clinical macrographic photographs of oral lesions with an accuracy of 85 − 91.56% to differentiate between malignant, pre-malignant and benign lesions^30,31^.

Quantitative analysis of non-invasive confocal microscopy images is a growing area of research^32^. Previous approaches for analysing confocal microscopy imaging using exogenous fluorescence intensity and autofluorescence spectrum have yielded variable results based on their respective bespoke algorithms^33,34^. Dittberner et al. 2016 developed an approach for detection OSCC in confocal microscopy images by calculating the distance between cell borders using a custom algorithm, obtaining an accuracy of 74%^35^. Jaramenko et al. (2015) and Aubreville et al. (2017) tested the efficacy of feature-based, patch-based and transfer learning CNN methods to identify OSCC, with a classification accuracy ranging from 70.6 to 87.02%^36,37^. One of the CNN methods explored by Aubreville et al. on their confocal video sequences from 12 subjects was transfer learning on Inception_v3 with an accuracy of 87.02%^37^. The Inception_v3 models developed in our study showed a detection accuracy of up to 90% and 94% for OED and OSCC respectively with other metrics to provide a well-rounded understanding of the results^10^.

While several CNN studies provide accuracy as a performance metric, it has several limitations such as overestimating model performance in cases of class imbalance in data samples and overlooking prediction distribution across classes^38^. Precision and specificity metrics can be particularly useful when false positives of non-dysplastic tissue being predicted as oral cancer lesions could impact the patient treatment experience negatively. This is balanced by the sensitivity metric, which is critical in the interpretation of false negative predictions. Potential undiagnosed OPMDs and OSCC resulting in disease advancement without timely intervention could be prevented by having a high sensitivity. The F1 score as a harmonic mean of precision and sensitivity balances the forementioned metrics for imbalanced class datasets similar to those described in the current study. To complete the model assessment, AUC-ROC provides a threshold-independent measure of the model’s ability to discriminate between classes by also taking model confidence into account^39^. Future studies could consider utilising data augmentation techniques or resampling methods (such as SMOTE) to address class imbalance issues.

To further encourage generalisability in the CNNs developed, grid search hyperparameter optimisation was utilized. While grid search is an exhaustive, thorough method, it could result in heavy computational costs and time as the parameter search space is widened. Other methods such as the Bayesian optimization algorithm could be considered to build a probabilistic model that predicts the parameters to test^40^. However, due to its complexity of implementation and a tendency to occasionally perform worse than grid search algorithm, it was not utilized in the current study^12^.

The number of epochs was optimized as it closely relates to overfitting and underfitting biases. There is no prescribed number of epochs that can be applied to every deep learning situation due to variations in size, shape, quality, and complexity of datasets and this value is often chosen using heuristics and trial and error^9^. Overfitting occurs when an algorithm focuses too much on minimizing errors by memorizing specific training examples, including noisy or irrelevant features, instead of learning the underlying patterns leading to lack of generalisability on unseen data^41^. Underfitting occurs when an algorithm fails to capture the underlying structure of the data resulting in high bias and poor performance on unseen data^42^. To attempt mitigation of overfitting and underfitting in the present study, a range of values for number of epoch were applied using a grid search algorithm.

Learning rate is a numerical factor chosen by machine learning practitioners that determines the step size at which the model parameters update during each iteration of training in the stochastic gradient descent algorithm^9^. The learning rate value applies a multiplier to changes made to the internal parameters by the SGD algorithm^43^. A higher learning rate means larger updates to the parameters that can lead to faster convergence but may also risk overshooting the optimal solution or causing instability. Conversely, a lower learning rate results in smaller updates, leading to slower convergence but potentially requiring more computation resources^44^. Grid search was used to optimise the learning rates for the models in this study. Despite the confocal microscopy images belonging to the same imaging modality, the optimal hyperparameter combinations for all three CNNs developed in this study were different. Applying one set of hyperparameters to all models would have resulted in a suboptimal model. This highlights the further need for hyperparameter optimization techniques in oral medicine deep learning research^45^.

While the classification performance of the quality filtering QMR model was high, it was not the same for all intraoral locations, with non-keratinized tissue images easier for the model to correctly classify than keratinized tissue (Table 3). One possible explanation could be the difference in the penetration of 480 nm incident laser of the confocal microscope in tissues with different properties. Considering the tongue, floor of the mouth, alveolar mucosa, soft palate, and buccal mucosa are some of the more common sites for OSCC, it is promising that the QMR model had close to 90% accuracy in filtering out diagnostic quality images from those sites. Overall, only 21.6% of the originally captured micrographs were of diagnostic quality based on the classification of the best ranked QMR model. This highlights the difficulty in acquiring diagnostic quality images in the oral cavity due to the interference of saliva, motion artifacts due to patient movement during capture, and intra-oral sites that are difficult to access with the handheld imaging probe^16^.

The challenge with classifying OPMDs is that they comprise a range of clinically recognized conditions, each with a varying degree of risk for developing into OSCC^5^. Grading the microscopic identification of OED can aid in management strategies and there are multiple grading systems^6^. A systematic review by de Freitas Silva et al. (2021) found no evidence to suggest that either the binary or the WHO histologic grading systems are better at predicting malignant transformation. However, the review found better inter-observer agreement in the binary system^46^. While the binary system has been reported to have better reproducibility^47^, the WHO 2017 OED grading system may be superior in predicting malignant transformation^48^. The consensus that simplifying dysplastic grading improves intra and inter-observer variability was the basis of the binary OED grading system, and therefore this was used in the present study and adopted in the PMAC diagnostic triage classification system^49^.

Once the images were filtered and assessed through the respective acriflavine and fluorescein diagnostic models for training and testing, it was evident that the FPMAC model performed better overall despite having a smaller training and test dataset (Fig. 5). The APMAC model had low clinical utility despite having a high classification performance for identifying lichenoid and low-risk lesions, since it failed to differentiate histopathologically non-dysplastic oral mucosa from OSCC and high-grade dysplasia (Fig. 5). However, the identification of low-risk and lichenoid lesions can be an important step in the early detection and monitoring of potentially cancerous lesions. Thus, imaging acriflavine stained in vivo tissue as part of diagnostic triage is potentially informative. In contrast, the FPMAC model showed high classification accuracy and AUC (> 0.9) for all 4 disease categories, with high discriminative power and robustness in identifying lichenoid lesions, low-risk and high-risk OED lesions and OSCC. This high performance was augmented by the incredibly rapid classification speed at the rate of 0.04 seconds per image.

Both APMAC and FPMAC diagnostic models exhibited strong performance on images from the floor of the mouth suggesting the characteristics of the epithelium with access to the location aided in model performance. FPMAC’s performance on the soft palate was exceptional, indicating the influence of the fluorescein contrast agent at this site on CNN diagnoses. The models’ poor performance on the gingiva, vestibule, hard palate and tongue sites suggested that these regions pose greater challenges for diagnostic identification using this technology. This could be linked to tissue keratinisation, imaging artifacts due to difficulty in access or movements of the patient while imaging, or lack of representation of characteristic disease specific signs captured in the imaging. The variation in performance of CNNs highlights the importance of evaluating model performance on a diverse set of locations.

The contrast agents acriflavine and fluorescein have a different tissue localization associations, the former localising to cell nuclei and the latter highlighting the entire pan-cytoarchitectural space. Such differential staining patterns may be relevant to the identification of certain epithelial landmarks observed in OED and OSCC. Lesions with differing levels of epithelial dysplasia may show architectural changes such as irregular epithelial stratification, increased nuclear-cytoplasmic ratio, and hyperchromatic nuclei among other features^5^. In contrast, OLP and OLL may demonstrate a band-like lymphocytic infiltrate, basal cell degeneration, and saw-toothing of the rete ridges^18^. Whilst OSCC is characterised by significant epithelial architectural and cellular changes in conjunction with invasion of malignant squamous cells with a desmoplastic stromal response^50^. The use of acriflavine may result in nuclear features such as hyperchromatic nuclei and increased nuclear-cytoplasmic ratio being more apparent. However, it has the potential to miss other cellular and architectural changes present in the cytoplasm or extracellular matrix, which may be critical for diagnosis. Fluorescein on the other hand tends to provide better identification of abnormalities in epithelial stratification and stromal interactions which may be key to differentiating between low- and high-risk OEDs, OLP/OLL and OSCC.

While fluorescence confocal microscopy can provide valuable data, the acquisition of these images has a few known challenges^32^. Stabilizing the handheld confocal microscope poses a significant challenge, making it difficult to accurately target lesions with the handheld probe. This issue is further compounded by artifacts caused by patient breathing movements and the accumulation of mucus or blood on the optical probe. Additionally, the reliability of imaging quality depends on the imaging practitioner’s knowledge and experience with handling specific confocal microscopy tools^51,52^. Additionally, the data collected could introduce new biases while being used to train deep learning models. The limited sample size and lack of population diversity in training datasets pose significant challenges particularly in ensuring fairness, accuracy, and generalization across global populations. Unrepresentative data can embed biases that disproportionately affect under-represented groups, reducing the model’s reliability and equity^53^. This issue is especially critical in fields like healthcare, where diagnostic tools must perform consistently across diverse demographics. Addressing these challenges requires the collection of more representative datasets, use synthetic data augmentation, and implementing global data sharing and collaboration to ensure that deep learning models are both inclusive and effective^54^.

The models developed in this study show promising outcomes in terms of identifying lichenoid lesions, low-grade OED, high-grade OED, and OSCC in an outpatient setting using in vivo microscopy images. The analysis pipeline from topically staining the region of interest to imaging the patient and receiving an AI-powered diagnostic report could be executed in less than 5 minutes in a clinical setting. The non-invasive early detection of OPMDs and oral cancer using such diagnostic analysis deep learning algorithms has the potential for a major positive impact on treatment outcomes for lesions that undergo malignant transformation^55^.

## Conclusions

The highly accurate QMR CNN as a quality filter rapidly provides meaningful data with real-time instantaneous image quality filtering. This important step can be harnessed to provide real-time image quality feedback, reducing time to diagnostic quality image capture. The diagnostic CNN models developed in the present study clearly demonstrate the ability to provide accurate diagnostic triage of oral mucosal conditions analogous to histopathological diagnosis relevant to current clinical practice. This facility will reduce the number of required scalpel biopsies by correctly identifying the majority of the oral epithelial dysplastic lesions. Additionally, the selection of appropriate hyperparameters for training deep learning models is a decision that can vastly impact the model performance.

## Electronic supplementary material

Below is the link to the electronic supplementary material.

Supplementary Material 1

## Data Availability

The raw image data collected from the participants in this study are stored on the University of Melbourne private online cloud storage. Patient data is both private and sensitive, therefore it can only be made available upon special request and consideration of the university.All python and PyTorch code used for the development of the deep learning models described in this study are freely accessible from an online GitHub repository: https://github.com/Rira-zen/QMR_PMAC_paper.
