# Supplementary material for: Convolutional neural networks for accurate real-time diagnosis of oral epithelial dysplasia and oral squamous cell carcinoma using high-resolution in vivo confocal microscopy
Source: Sci Rep. 2025 Jan 20;15:2555. doi: 10.1038/s41598-025-86400-5 (PMC11746977; doi:10.1038/s41598-025-86400-5)
Supplement: Supplementary file 1 — Supplementary Material 1 [file 41598_2025_86400_MOESM1_ESM.docx]

| **Supplementary Table 1:** Images retained and rejected by the QMR quality filtering CNN | | | | |
| --- | --- | --- | --- | --- |
| **Intra-oral locations** | **Acriflavine** | | **Fluorescein** | |
|  | **Retained -  n (%)** | **Rejected – n (%)** | **Retained – n (%)** | **Rejected – n (%)** |
| Buccal Mucosa | 277 (33.05) | 561 (66.95) | 125 (18.83) | 539 (81.17) |
| Floor of Mouth | 101 (21.04) | 379 (78.96) | 58 (14.11) | 353 (85.89) |
| Gingiva and Vestibule | 355 (21.39) | 1305 (78.61) | 186 (16.96) | 911 (83.04) |
| Hard palate | 21 (14.58) | 123 (85.42) | 2 (2.33) | 84 (97.67) |
| Soft palate | 35 (47.30) | 39 (52.70) | 39 (51.32) | 37 (48.68) |
| Tongue | 554 (25.61) | 1609 (74.39) | 230 (15.59) | 1245 (84.41) |
| **Grand Total** | **1343 (25.06)** | **4016 (74.94)** | **640 (16.80)** | **3169 (83.20)** |

| **Supplementary Table 2:** Image distribution across both datasets based on intra-oral location | | | | |
| --- | --- | --- | --- | --- |
| **Intra-oral locations** | **Acriflavine** | | **Fluorescein** | |
|  | **Training** | **Test** | **Training** | **Test** |
| Buccal Mucosa | 220 | 57 | 97 | 28 |
| Floor of Mouth | 86 | 15 | 50 | 8 |
| Gingiva and Vestibule | 283 | 72 | 153 | 33 |
| Hard palate | 18 | 3 | 2 | 0 |
| Soft palate | 25 | 10 | 30 | 9 |
| Tongue | 449 | 105 | 183 | 47 |
| **Grand Total** | **1081** | **262** | **515** | **125** |
